# PACAP regulates neuroendocrine and behavioral stress responses via CRF-containing neurons of the rat hypothalamic paraventricular nucleus

**DOI:** 10.1038/s41386-024-02016-9

**Published:** 2024-10-29

**Authors:** Karl Ebner, Veronica Fontebasso, Federico Ferro, Nicolas Singewald, Jens Hannibal

**Affiliations:** 1 Department of Pharmacology and Toxicology, Institute of Pharmacy and Center for Molecular Biosciences Innsbruck, University of Innsbruck, Innsbruck, Austria; 2 Faculty of Health and Medical Sciences, Institute of Clinical Medicine, University of Copenhagen, Copenhagen, Denmark; 3 Department of Clinical Biochemistry, Faculty of Health Sciences, Bispebjerg and Frederiksberg Hospital, University of Copenhagen, Copenhagen, Denmark

**Keywords:** Stress and resilience, Depression, Neurotransmitters, Predictive markers, Anxiety

## Abstract

Pituitary adenylate cyclase-activating polypeptide (PACAP) is a neuropeptide widely distributed in the brain including the hypothalamic paraventricular nucleus (PVN) implying a regulatory role in stress function. Recent evidence indicates that one of the main targets of PACAP within the PVN are corticotropin-releasing factor (CRF) neurons, which are key regulators of the hypothalamic-pituitary-adrenal (HPA) axis. However, the neural correlates that mediate PACAP effects on stress function are not fully understood. In the present study, we characterized the neuronal mechanism by which PACAP regulates neuroendocrine and behavioral stress responses in rats. We found that intracerebroventricular administration of PACAP increased the swim stress-induced c-Fos expression in distinct brain areas of the stress and anxiety circuitry including the parvocellular part of the PVN and changed behavioral stress coping during forced swimming to a more passive coping style (i.e., indicated by increased floating and reduced struggling behavior). Subsequently, PACAP administration directly into the PVN mimicked these behavioral effects and potentiated the plasma ACTH response to forced swim stress suggesting an excitatory role of PACAP on HPA stress axis reactivity. In addition, immunohistochemical analysis revealed a considerable portion of stress-activated CRF neurons in the medial parvocellular part of the PVN that co-localized PAC1 receptors suggesting that PACAP-induced effects on stress function are likely mediated directly by activation of CRF neurons in the PVN. Thus, these findings suggest that the PVN may represent one of the key areas where PACAP regulates the neuroendocrine and behavioral stress response.

## Introduction

Pituitary adenylate cyclase-activating polypeptide (PACAP) is a neuropeptide belonging to the secretin/glucagon/vasoactive intestinal peptide (VIP) family [1]. It was originally isolated from ovine hypothalamic tissue where it was demonstrated to activate adenylate cyclase and elevate cAMP levels in perfused anterior pituitary cells [2]. In mammals, PACAP is present in two amidated forms, PACAP27 and PACAP38, that are derived from the same precursor protein encoded by the *Adcyap1* gene [3]. However, tissue measurements have revealed that PACAP38 is by far the predominant form in the mammalian brain and PACAP27 represents less than 10% of the total peptide content [3]. PACAP exerts its effects mainly via its cognate PAC1 receptor, which binds PACAP with an affinity of 1000-fold greater than VIP. However, the two VIP-preferring receptors (VPAC1 and VPAC2 receptors) bind PACAP and VIP with equal affinities [3, 4]. In the brain, PACAP and PAC1 receptors are highly expressed in areas implicated in stress and anxiety regulation including the hypothalamus, amygdala and brainstem [5–8]. On the other hand, PACAP administration into stress-associated structures such as amygdala, bed nucleus of the stria terminalis (BNST) or prefrontal cortex has been shown to produce stress-like responses including the activation of the hypothalamic-pituitary-adrenal (HPA) axis [9–15]. Conversely, the exposure to acute or chronic stressors increases PACAP expression within these areas [16–18]. Furthermore, PACAP or PAC1 receptor-deficient mice show a dysregulated corticosterone circadian rhythm, diminished stress-induced HPA axis activation [19–23], and reduced anxiety-like behavior and fear [21, 24–27]. Thus, these data suggest that PACAP signaling may serve as an important regulator of the stress and anxiety response, and may contribute to the pathogenesis and maintenance of stress-related psychopathologies (for review see refs. [28–31]).

In humans, a single nucleotide polymorphism of the PACAP and/or PAC1 receptor gene has been related to psychiatric disorders such as depression [32, 33], anxiety disorders and post-traumatic stress disorder (PTSD) [34–36]. Moreover, genetic variants in the PACAP/PAC1 genes were associated with altered treatment efficacy in generalized anxiety disorder [37] and circulating PACAP levels were positively correlated with anxiety disorders [35, 36, 38], PTSD symptom severity, and diagnostic status [35] or intrinsic amygdala functional connectivity in PTSD [38]. Thus, human studies suggest translational evidence for a dysregulation of the PACAP system and implicate PACAP as a useful biomarker for the severity of psychiatric symptoms in response to psychological stressors.

However, the exact neuronal mechanisms through which PACAP mediates its effects on physiological and behavioral stress functions are still poorly understood. The hypothalamic paraventricular nucleus (PVN) is an integrative center in the brain orchestrating a wide range of endocrine, autonomic, and behavioral responses to stress [39–41]. Notably, within the brain the highest concentration of PACAP nerve fibers and PAC1 receptors are present in the PVN, especially within the medial parvocellular subdivision of the PVN where corticotropin-releasing factor (CRF) neurons, the main regulators of the neuroendocrine stress response, are localized [5–7, 42–46]. Electron microscopy revealed the presence of synapses between PACAP-containing terminals and CRF-perikarya and dendrites in the parvocellular PVN [44]. Central PACAP administration elevates c-Fos expression (a marker of neural activation) and CRF gene expression in the PVN [9, 47, 48]. Further, PACAP administration stimulates phosphorylation of the transcription factor CREB in PVN-CRH neurons and elevates plasma ACTH/corticosterone levels [9, 49] suggesting that PACAP regulates HPA function through mechanisms within the PVN. However, as these studies were conducted only under basal conditions, the specific role of the hypothalamic PACAP/PAC1 receptor system under stress conditions is still not known.

Thus, the aims of the current study to further characterize the functional role of central PACAP during the stress response in rats were threefold. First, we investigated the effect of intracerebroventricular (ICV) PACAP38 administration on stress-related behavior in the forced swimming, a commonly used behavioral challenge for eliciting a stress response and assessing stress-coping behavior in rodents [50, 51]. Second, to identify neuronal correlates influenced by PACAP, we assessed the neuronal responsiveness to forced swim stress by quantifying the c-Fos expression in selected forebrain areas of the stress and anxiety circuit including the PVN. Third, we examined the effects of intra-PVN infusion of PACAP38 on behavioral and neuroendocrine response to swim stress and examined the distribution and localization of PAC1 receptors in putative stress-activated neurons within the PVN.

## Materials and methods

Detailed “Materials and methods” can be found in the Supplementary Material.

### Animals

All experimental procedures on adult male Sprague–Dawley rats were approved by the National Ethical Committee on animal care and use (Bundesministerium für Bildung, Wissenschaft und Forschung) in compliance with international laws and policies.

### Surgery

All surgical procedures were performed under sterile conditions as previously described [52, 53]. Animals were placed in a stereotaxic frame (Stoelting, Illinois, USA) and a 23-gauge stainless steel guide cannula (15 mm length; o.d. 0.64 mm, i.d. 0.34 mm; Injecta GmbH, Germany) was implanted either unilaterally 1 mm above the right lateral ventricle (coordinates from bregma: AP 0.8 mm, ML + 1.4 mm and DV −3.0 mm) or bilaterally 2 mm above the left and right PVN (coordinates from bregma: AP 1.9 mm, ML ± 1.7 mm, DV −6.3 mm, with an angle of 10°) by using a rat brain atlas [54]. During recovery single housed rats received analgesic care and the locations of the cannula track were histologically verified after experiments.

#### Implantation of a jugular venous catheter

A silastic-tipped vinyl catheter was inserted into the left jugular vein, routed under the skin and exteriorized at the neck of the animal as described previously [52]. Blood sampling through a pre-implanted jugular venous catheter allows repeated blood sampling from conscious, freely moving rats without restraining animals.

### Forced swim challenge

The forced swim challenge was conducted as described previously [55]. Briefly, rats were placed individually in a square plastic tank (35 × 35 cm) filled with water (20 ± 1 °C) to a 30-cm depth. During the forced swimming session, the behavior was recorded by a video system for subsequent analysis. Animal’s behavior was scored by a trained observer blind to the treatment of animals, quantifying absolute time measurements (struggling, swimming, and floating) [55]. After the 5-min swimming session, animals were gently dried using a towel and returned to their home cage.

### Drug microinjection procedure

For ICV infusions, rats were gently held inside their cages and the stylets of guide cannulas were removed. Drugs were injected into the right lateral ventricle (1.5 µl/rat) over a period of approximately 1 min using a 5-µl syringe (Hamilton Instruments, Switzerland) that was connected via a polyethylene tubing to a 30-gauge injection cannula (tip extending 1 mm beyond the guide). The internal cannula was held in a position for another 2 min after injection to allow diffusion of drugs before being slowly withdrawn. Rats were returned to their home cage and 15 min after the injection exposed to the modified forced swim test.

For bilateral microinfusions into the PVN, stylets of guide cannulas were replaced by two 30-gauge microinjection cannulas that were 2 mm longer than the guide cannulas, thus reaching the PVN. Injection cannulas were connected to a 5 cm long PE-10 tubing and filled with drug or vehicle solution. This infusion device was connected to a syringe mounted on a microinfusion pump (TSE-Systems, Bad Homburg, Germany) via a 100 cm long polyethylene tubing interconnected with a dual channel fluid swivel system (Instec Laboratories, Boulder, USA) and was installed approximately 1 h before starting experiments. Dugs were infused over a period of 7.5 min at a defined flow rate of 0.2 µl/min (1.5 µl/injection side) without any stressful manipulations (e.g such as capturing or restraining animals) before and during the infusion procedure. After the microinjection procedure, the injection cannula was left in the guide cannula for another 2.5 min before being removed. Thereafter, animals were exposed to the forced swim stressor.

### Blood sampling and ACTH measurements

After a 60-min habituation, the experiment started with the collection of two blood samples (0.3 ml) under basal conditions, 35 and 15 min prior to stress exposure. Bilateral infusions of drugs into the PVN started 12 min before the onset of the stressor. After injections, another blood sample was taken and 1 min later animals were exposed to the forced swim stress procedure for 5 min. During the forced swim session, behavioral output was scored and analysed as described above. After animals were returned to their home cages three additional blood samples were collected at regular intervals (10, 30, and 60 min after the onset of the stressor). Sampled blood volumes were immediately replaced with an equal volume of heparinized saline. Blood was collected into EDTA-coated tubes containing 10 units of sodium heparin (Wako Pure Chemical Industries Ltd., Osaka, Japan), placed on ice and immediately centrifuged at 3000 g for 10 min at 4 °C. Supernatants were collected and stored at −80 °C until measurement. Plasma ACTH concentrations were determined using a commercially available immunoassay kit (MP Biomedicals, Orangeburg, NY, USA) according to the manufacturer’s protocol. The intra- and inter-assay coefficients of variation were below 7 and 10%, respectively.

### Drugs

PACAP38 (Bachem AG, Switzerland) were dissolved in sterile distilled water and aliquots of stock solution (1 mg/mL) were stored at −80 °C. For preparation of the working solution the liquid of concentrated stock solution was diluted with artificial cerebrospinal fluid (aCSF; 140 mM NaCl, 3.0 mM KCl, 1.25 mM CaCl_2_, 1.0 mM MgCl_2_, 1.2 mM Na_2_HPO_4_, 0.3 mM NaH_2_PO_4_, 3.0 mM glucose and pH adjusted to 7.4) to a final concentration of 10 or 100 µM. Vehicle animals received a 1.5 µl infusion of aCSF solution. All drugs were freshly prepared before each experiment and kept on ice during the experimental procedures.

### Histological verification of cannula placements

Verifications of cannula placements were made before analyzing neuroendocrine and behavioral experiments under a microscope on the basis of previous definitions of a brain atlas [54]. Only data from rats found to have injector tracks extending into appropriate target sites were included in the analyses.

### Perfusion and brain tissue processing

Two hours after stress exposure, animals were deeply anesthetized with sodium thiopental (250 mg/kg i.p., Sandoz, Austria) and transcardially perfused with 300 ml of 0.9% saline followed by 300 ml of fixative (either 4% paraformaldehyde in 0.1 M PBS or 2% paraformaldehyde with 0.2% picric acid in 0.1 M PBS, Stefanini´s solution). Brains were removed and kept in fixative at 4 °C overnight. After post-fixation, the brains were washed twice in 0.2 M PBS (c-Fos immunohistochemistry) or equilibrated in 0.05 M PBS containing 30% sucrose for 48 h at 4 °C (Immunofluorescence staining) until further processing. Series of 40-µm-thick coronal sections of respective brain areas were sectioned by using either a vibratome (Leica VT1000S, Leica Microsystems, Germany) or a cryostat (Leica CM1950, Leica Microsystems, Germany). The sections were stored in a cryoprotectant solution at 4 °C until further processing.

### Immunohistochemistry and immunofluorescence staining

Immunohistochemistry was performed on free-floating brain slices (40 µm) using rabbit anti c-Fos primary antibody (Santa Cruz Biotechnology) as described previously [52]. Cells containing a nuclear brown-black 3,3′-diaminobenzidine (DAB) staining were considered as c-Fos positive cells. The reaction was terminated once an optimal contrast between specific cellular and nonspecific background labeling was reached. Immunoreactive cells were quantified manually in the regions of interest by a trained experimenter blind to the treatment groups. The total number of c-Fos-positive cells from each brain region were quantified bilaterally from 1–2 sections per rat in a tissue area of 0.01 mm^2^ using a brightfield microscope (Olympus BX-51, Olympus Corporation) equipped with a computer-assisted image analysis system. Sections from all investigated animals were chosen (based on neurochemical and functional criteria of distinct subregions, see Supplementary Material for further details) at identical rostro-caudal levels, making direct comparison between animals possible. Anatomical localization of selected brain regions was aided by using illustrations of a rat brain atlas [54].

Immunofluorescence staining and photomicroscopy was performed as previously described [56] (see Supplementary Material for further details).

### Statistical analysis

Experimental subjects were included in the statistical analysis only if the microinjection cannulas were confirmed to be localized in the respective brain region. Statistical analysis was performed using GraphPad Prism 5. Counts of c-Fos positive nuclei within the different brain areas and behavioral data after ICV drug injection were analyzed using parametric Student’s two-tailed *t*-test. Behavioral data from more than two groups were analyzed by one-way ANOVA followed by Dunnet's multiple comparison post hoc test. Plasma concentrations of ACTH were analyzed by two-way ANOVA (treatment × time) with repeated measures on the last factor followed by appropriate post hoc analysis. Data are presented as mean ± standard error of the mean (SEM). In all cases, *P* < 0.05 was considered to be statistically significant.

## Results

### Effect of ICV PACAP38 administration on behavioral responses to forced swim stress

**Fig. 1:**
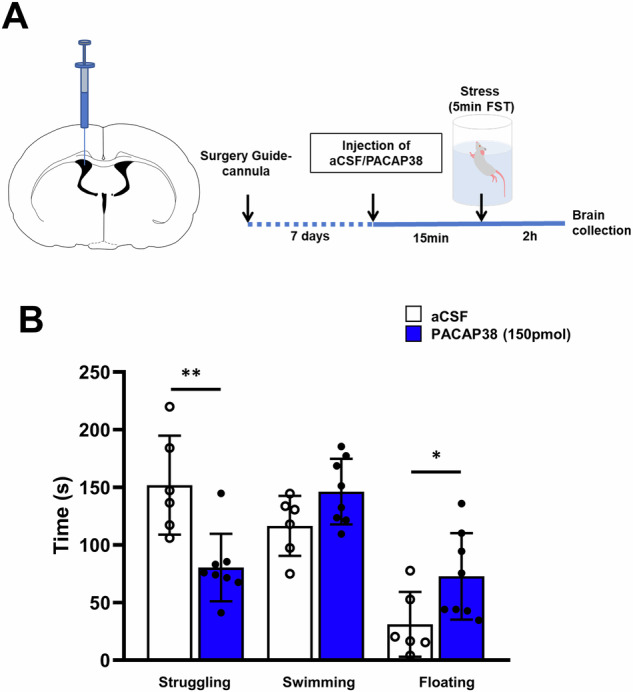
Effects of ICV PACAP38 administration on the behavioral stress response of rats exposed to the modified forced swim test. **A** A schematic coronal section from the rat brain atlas [54] illustrating cannula implantation into the right lateral ventricle (left panel) and the experimental timeline (right panel) (**B**) PACAP38-injected animals (*n* = 8) showed a significant reduction in active coping behavior, as indicated by decreased struggling, and an enhancement of immobility (floating behavior) compared to aCSF-injected controls (*n* = 6). Data are expressed as mean ± SEM. *p < 0.05, **p < 0.01 vs aCSF-injected controls (Student's *t-*test unpaired).

We investigated the effect of central PACAP38 administration on the stress-coping behavior of rats during a 5-min forced swimming session (Fig. 1A). Male rats were randomly assigned either to the treatment or control group and administered either with PACAP38 (150 pmol) or vehicle (aCSF), respectively. However, we had to exclude one animal from the control group because of malfunctioning video tracking system during forced swim exposure. This resulted in a final *n* = 6 (vehicle) and *n* = 8 (PACAP38). As shown in Fig. 1B, PACAP38 injection significantly reduced struggling behavior (*P* < 0.001) and increased floating behavior (*p* < 0.05) indicating a more passive coping strategy in response to uncontrollable stress compared with vehicle-treated controls (Fig. 1B). Thus, this finding indicates that increased central PACAP function produces a reorganization of behavioral stress-coping to a more passive mode.

### Effect of ICV PACAP38 administration on neuronal activity in selected forebrain areas of animals subjected to forced swim stress

**Fig. 2:**
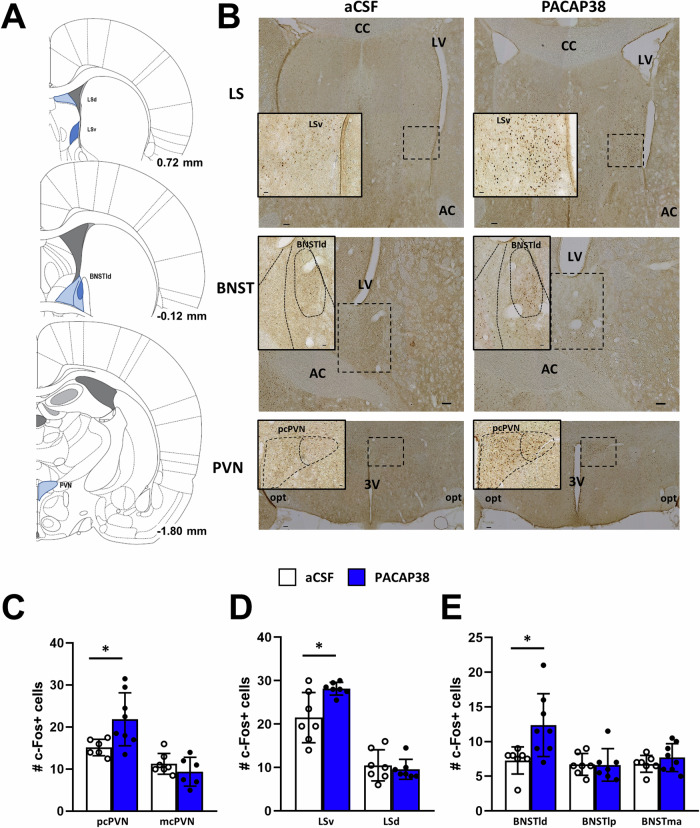
Effects of ICV PACAP38 administration on swim stress-induced c-Fos expression in selected brain regions. **A** Schematic coronal sections from the brain atlas [54] showing brain regions examined (gray shaded). **B** Representative photomicrographs showing c-Fos positive cells in rats exposed to forced swim stress and microinjected either with aCSF or PACAP38. The lower panels show c-Fos quantification presented as bar graphs in the PVN (**C**) LS (**D**) and BNST (**E**). There was a significant increase of stress-induced c-Fos expression in the parvocellular part of the PVN, ventral LS and latero-dorsal part of the BNST of PACAP38-treated animals compared to aCSF-injected controls. Abbreviations: 3 V third ventricle, AC anterior commissure, BNST bed nucleus of the stria terminalis, BNSTld latero-dorsal part of the BNST, BNSTlp latero-posterior part of the BNST, BNSTma medial-anterior part of the BNST, CC corpus callosum, LS lateral septum, LSd lateral septum dorsal, LSv lateral septum ventral, LV lateral ventricle, opt optic nerve, PVN paraventricular nucleus of the hypothalamus, pcPVN parvocellular part of the PVN, mcPVN magnocellular part of the PVN. *N* = 6–8 animals per group. Scale bars: 100 µm at lower magnification, 25 µm at higher magnification images. Data are expressed as mean ± SEM. *p < 0.05 vs aCSF-injected controls (Student’s *t-*test).

The effects of ICV administration of PACAP38 (150 pmol) or vehicle (aCSF) on swim stress-induced c-Fos expression as a marker of neuronal activity was visualized and quantified within six brain regions of the stress and anxiety circuitry. The brains of 15 animals were analyzed, whereby one animal had to be excluded from analysis due to tissue destructions in the respective brain area. Thus, in total a final *n* = 6 (controls) and *n* = 8 (PACAP38) were included in this study. The selected brain areas included the PVN (magnocellular and parvocellular part), the lateral septal nucleus (dorsal and ventral part), the amygdala (central, basolateral, and medial nuclei), and the BNST (latero-dorsal, latero-posterior, and medial-anterior part of the dorsal BNST). Anatomical regions of interest were identified using a rat brain atlas [54] and standard anatomical landmarks (e.g., third ventricle, fornix, optic tract) to define nuclear boundaries (Fig. 2A). Statistical analysis by Student's *t-*test revealed that ICV PACAP38 administration potentiated stress-induced c-Fos expression in the parvocellular part of the PVN (*t*(12) = 2.49, *p* = 0.02), but not in the magnocellular part, indicating a selective PACAP-induced hyperactivation within the site of the PVN, where CRF-expressing neurons are localized (Fig. 2B, C). Notably, swim stress-induced neuronal activation was increased by 46% in the parvocellular PVN of PACAP38-treated rats compared to controls. Moreover, PACAP38 also increased stress-induced neuronal activation in the ventral LS (*t*(12) = 2.94, *p* = 0.01; Fig. 2D) and dorsolateral part of the anterior BNST (*t*(13) = 2.749, *p* = 0.01; Fig. 2E) indicated by a 33 and 75% increase of stress-induced c-Fos expression in PACAP38-treated animals compared to controls. In all other brain regions examined, there was no statistical difference in swim stress-induced c-Fos expression between PACAP38 and vehicle-treated controls (Figs. 2 and S1). Thus, these findings demonstrate a selective PACAP-induced hyperactivation within distinct limbic areas and indicate these brain areas as potential sites where PACAP mediates its stress functions.

### Effect of ICV PACAP38 administration on basal c-Fos expression

In rats not exposed to the forced swim stress (basal condition), the number of cells expressing c-Fos protein was low in most areas examined (Table S1). No significant differences in basal c-Fos expression were observed between PACAP38 (*n* = 6) and vehicle-treated controls (*n* = 6) in nearly all brain regions investigated with the exception of the parvocellular part of the PVN. In this region, PACAP-injected animals exhibited significant higher c-Fos expression compared to controls (*t*(10) = 4.278, *p* = 0.01; Table S1).

### Effect of bilateral microinjection of PACAP38 into the PVN on behavioral and neuroendocrine responses to forced swim stress

**Fig. 3:**
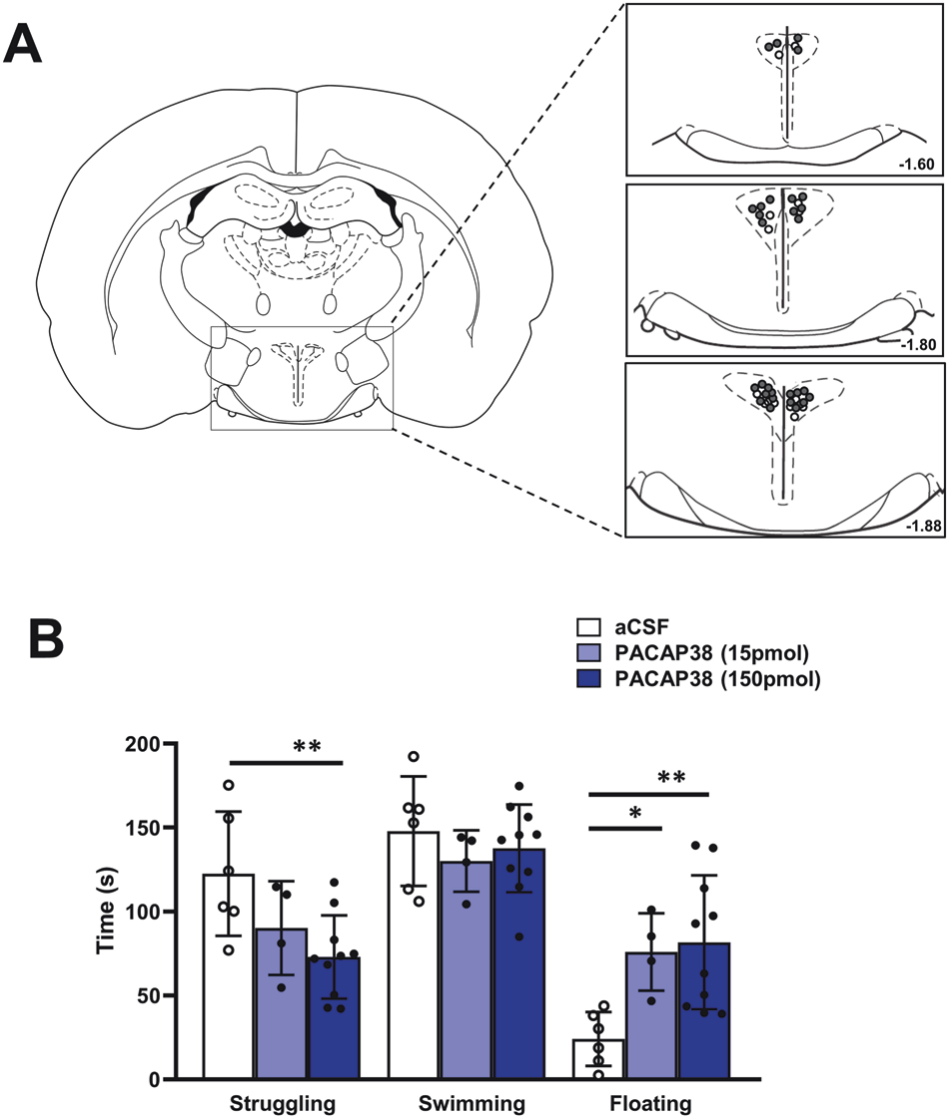
Effects of PACAP38 administration into the PVN on the behavioral stress response of rats exposed to the modified forced swim test. **A** Schematic drawings of coronal sections of the rat brain showing the localization of the cannula tips within the PVN from rostral (−1.6 mm) to caudal (−1.88 mm) for bilateral microinjection of vehicle (white circles) and PACAP38 (black circles). **B** Intra-PVN microinjections of PACAP38 (150 and 15 pmol/site; *n* = 10 and 4) elicited significantly higher floating (passive coping) and reduced struggling (active coping) time compared to aCSF-injected controls (*n* = 6). No significant effects were found on the swimming behavior between PACAP38-injected rats and controls. Data are expressed as mean ± SEM. *p < 0.05 **p < 0.01 vs aCSF-injected controls (one-way ANOVA followed by Dunnet's multiple comparison post hoc test).

Histological analysis revealed that PVN injections were made between 1.6 and 1.9 mm posterior to bregma. Only animals with both cannulas correctly placed in the PVN (Fig. 3A) were included in this study. In addition, two animals were excluded from the analysis due to the tracking system stopping during swim exposure, thus preventing behavioral analyses. This resulted in a final *n* = 6 (controls) and *n* = 14 (PACAP38; 150 pmol *n* = 10, and 15 pmol *n* = 4). To determine the specific role of hypothalamic PACAP on stress-related behavioral regulation, we compared stress-coping behavior during forced swimming in PACAP38-treated animals and controls. In this experiment, we found that intra-PVN administration of PACAP38 prior to forced swimming significantly affected the coping behavior (Fig. 3B). Statistical analysis by one-way ANOVA indicated a significant difference between groups in struggling (*F*(2, 17) = 5.33, *p* = 0.01) as well as floating behavior (*F*(2, 17) = 6.56, *p* = 0.007). Post hoc analysis revealed that intra-PVN administration of PACAP38 dose dependently (15 and 150 pmol) reduced struggling time (*p* < 0.01 with the higher dose of 150 pmol) and increased floating time (*p* < 0.01 with the dose of 150 pmol and *p* < 0.05 with 15 pmol). However, intra-PVN administration of PACAP38 had no effect on the swimming behavior (*F*(2, 17) = 5.33, *p* = 0.55). Thus, these findings demonstrate that exogenous PACAP infused into the PVN reduces active coping and escape-oriented behaviors during a stressful situation such as forced swim and promotes a more passive coping strategy.

**Fig. 4:**
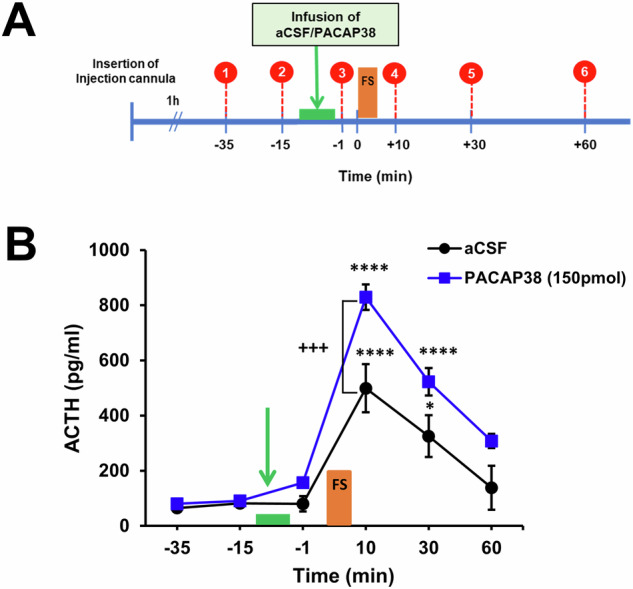
Effects of PACAP38 administration into the PVN on basal and swim stress-induced ACTH levels. **A** Schematic illustration of the experimental design with the timeline of blood sampling (red circles), drug infusion (green bar) and stress exposure (orange bar, forced swim, FS). Experiment started with insertion of the infusion device (bilateral injection cannulas connected to a microinfusion pump) at least 1 h before blood sampling started. Drugs were infused automatically at a constant flow rate over a period of 7.5 min without any stressful manipulations (e.g such as capturing or restraining animals) before and during the infusion procedure. Blood samples were collected at regular intervals before drug infusion under basal conditions (−35 and −15 min) and after drug infusion, but before stress exposure (−1 min) and after forced swim stress (10, 30, and 60 min). **B** Swim stress caused an increase in plasma ACTH levels in both intra-PVN PACAP38 (*n* = 7) and aCSF-injected controls (*n* = 7). Compared to controls, intra-PVN PACAP38-injected rats showed higher plasma ACTH levels during and after forced swim stress. However, basal levels did not differ between groups. The green bar indicates timing of intra-PVN infusion, the orange bar the forced swim (FS) stress exposure. Data are expressed as mean ± SEM. *p < 0.05 ****p < 0.0001 compared to basal timepoints (−35 and −15 min) in same treatment group; +++*p* < 0.001 compared to vehicle-injected controls at same timepoint (two-way ANOVA followed by Bonferroni's multiple comparison post hoc test).

To assess the impact of intra-PVN PACAP infusion on neuroendocrine stress response blood samples were collected at different time points before and after forced swim stress from the same cohort of rats that were bilaterally microinjected with PACAP38 or vehicle into the PVN (Fig. 4A). Moreover, four animals were excluded due to catheter blockages or obstructions preventing blood sampling procedure during experiment resulting in a final *n* = 7 (controls) and *n* = 7 (PACAP38, 150 pmol). As shown in Fig. 4, intra-PVN PACAP38 administration modulated the neuroendocrine stress response. The exposure to swim stress caused an increase in plasma ACTH levels in both PACAP38-treated animals and vehicle-treated controls (Fig. 4B). Statistical analysis of ACTH levels by two-way ANOVA revealed a significant effect of the main factors (treatment: *F*(1, 60) = 9.87, *p* = 0.0085; time: *F*(5, 60) = 69.34, *p* < 0.0001) as well as a significant interaction between main factors (*F*(5, 60) = 4.80, *p* = 0.0009). Subsequent post-hoc analysis indicated significant differences of ACTH levels between PACAP38-treated (150 pmol/site) animals and controls immediately after stress exposure with a higher ACTH response to forced swimming at 10 min (*p* < 0.003) after onset of the stressor in animals microinjected with PACAP38 compared to vehicle-injected controls. Thus, our data show that PACAP infused into the PVN causes a disinhibited plasma ACTH response to forced swim stress indicating a faciliatory effect on HPA axis stress response.

In a separate cohort of animals, we measured ACTH plasma levels in control animals without stress exposure over the entire time course of the experiment following intra-PVN PACAP38 administration. Our findings show that ACTH levels did not significantly differ between PACAP38-treated animals (*n* = 4) and controls (*n* = 5) at any point during the experiment, indicating that PACAP38 did not induce a sustained rise in ACTH levels in the absence of stress (Fig. S2).

### Distribution of PAC1 receptors in the PVN and colocalization with c-Fos and CRF

**Fig. 5:**
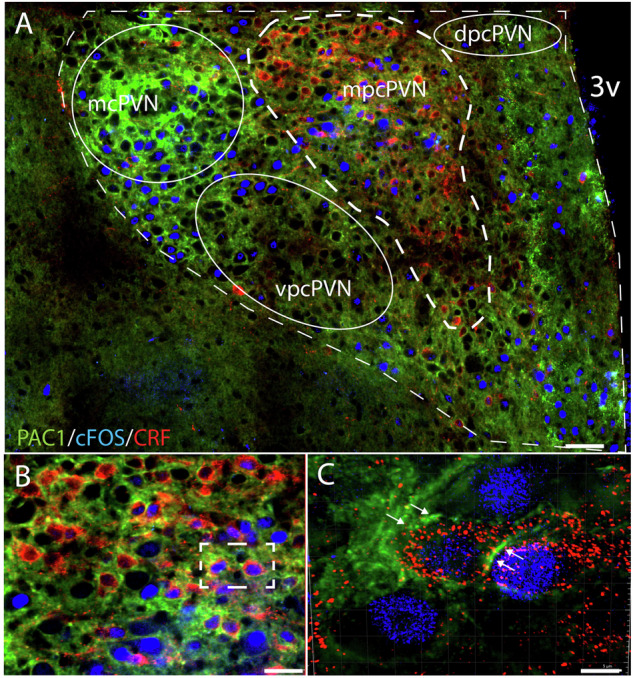
Distribution of PAC1 receptors in the PVN. Representative immunofluorescence photomicrograph from a rat exposed to forced swim stress showing PAC1 (green), c-Fos (blue) and CRF immunolabeling (red) in the PVN. **A** PAC1 receptors are widely distributed in different subareas of the PVN including the magnocellular (mcPVN) and parvocellular part (pcPVN). Moreover, PAC1 receptor immunoreactivity was found on stress-activated (c-Fos positive) neurons in both the magnocellular and parvocellular part. Notably, within the medial parvocellular part of the PVN (mpcPVN) a considerable portion of PAC1 positive neurons are also labelled for c-Fos and CRF (triple-immunofluorescence staining) indicative for a functional role of PAC1 receptors within this area. **B** shows representative triple-labelled neurons within the mpcPVN at higher magnification. **C** The insert in (**B**) in higher magnification (3D reconstructed deconvoluted imaged) demonstrating two CRF-containing neurons which are c-Fos positive (blue nuclei) and the CRF-expressing neurons express the PAC1 receptor in their membrane (green) as indicated by arrows. Scale bars: (**A**), 100 µm; (**B**), 25 µm; (**C**), 10 µm.

Immunofluorescence in combination with neuronal activation mapping was used to determine the colocalization of PAC1 and c-Fos in the PVN of stressed rats. Therefore, a separate cohort of animals was ICV administered with aCSF (controls, *n* = 3) or PACAP38 (150 pmol, *n* = 4) and exposed to swim stress. A representative immunofluorescence photomicrograph from a rat exposed to forced swim stress shows that PAC1 receptors are widely distributed in different subareas of the PVN including the magnocellular and parvocellular part (Fig. 5A). Moreover, in both subareas intense PAC1 receptor immunoreactivity was found on stress-activated (c-Fos positive) neurons. Notably, quantification of PAC1 receptor immunoreactivity in immunofluorescence images using a confocal 8-bit grayscale images calibration revealed no significant differences in PAC1 receptor immunoreactivity between PACAP38-treated animals and controls in either of the PVN subregions (Fig. S3). Additionally, we found no significant difference in PAC1 expression between the magnocellular and parvocellular part of the PVN (Fig. S3). These results suggest that PAC1 receptor distribution is similar across these PVN subregions and is not significantly altered by PACAP38 treatment under the conditions studied.

**Fig. 6:**
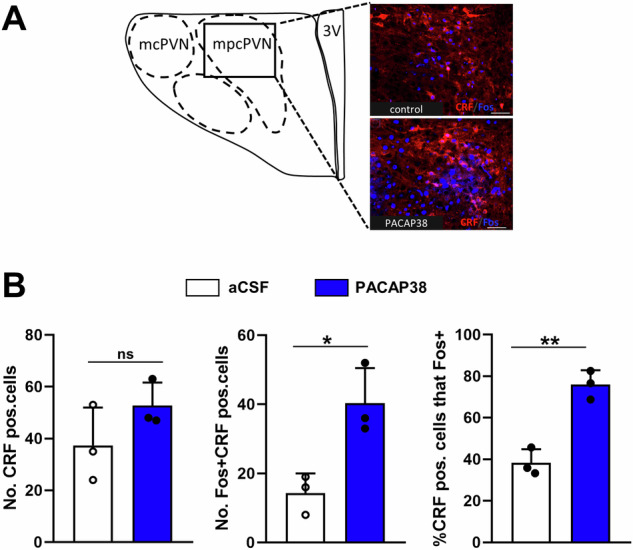
Quantification of CRF and c-Fos positive neurons in the medial parvocellular PVN (mpcPVN) of rats that were ICV injected with aCSF (controls) or PACAP38 (150 pmol) 15 min before swim stress exposure. **A** Schematic drawing illustrating different subregions of the PVN and representative confocal images showing CRF (red) and c-Fos (blue) immunopositive neurons in the mpcPVN. **B** The lower panels show quantification of CRF (left panel) and CRF/c-Fos (middle and right panel) positive neurons presented as bar graphs in the mpcPVN. There was no significant difference in the total number of CRF neurons between PACAP38-treated animals and controls, but the number of CRF neurons that co-express c-Fos were significantly higher in the PACAP38-treated animals compared to controls. Note that in controls only 38% of CRF neurons expressed Fos, while in the PACAP38-treated animals the percentage of CRF neurons expressing c-Fos was 76%. Abbreviations: 3 V third ventricle, ns not significant. *N* = 3 animals per group. Scale bar: 50 µm. Data are expressed as mean ± SEM. *p < 0.05 **p < 0.01 vs aCSF-injected controls (Student's *t-*test).

Moreover, we have conducted additional quantification of the confocal immunofluorescence data and analyzed the CRF and c-Fos expression in the medial parvocellular part of the PVN. Our data show that ICV PACAP administration significantly increased stress-induced c-Fos expression specifically in CRF neurons in this subarea of the PVN (Fig. 6). While the total number of CRF neurons did not differ between the PACAP38-treated and control group, the number of CRF neurons co-expressing c-Fos was significantly higher in the PACAP38-treated animals compared to controls. Notably, in control animals, only 38% of CRF neurons expressed c-Fos, whereas in PACAP38-treated animals, 76% of CRF neurons were c-Fos positive (Fig. 6). This suggests that PACAP exerts a critical effect in the medial parvocellular PVN under stress conditions, specifically through the activation of CRF-expressing neurons.

In order to establish whether PAC1 receptor distribution has a direct anatomical link with stress-activated CRF neurons in the parvocellular part of the PVN, we performed a triple-immunofluorescence staining of PAC1, c-Fos and CRF. Notably, within the medial parvocellular part of the PVN a considerable portion of PAC1 positive neurons are also labelled for c-Fos and CRF (triple-labelled neurons, Fig. 5B, C) indicative for a functional role of PAC1 receptors within this area.

## Discussion

The present study indicates that PACAP promotes a passive behavioral coping style and stimulates stress-related HPA axis function likely through a CRF-mediated mechanism in the hypothalamus. Specifically, we found that central administration of PACAP38 increases the stress-provoked neuronal activity (indicated by c-Fos expression) in brain areas associated with stress and anxiety functions such as PVN, LS, and BNST. In addition, ICV PACAP38 administration alters stress-coping behavior during forced swimming as PACAP-injected animals show increased passive (floating) and reduced active coping (struggling). These behavioral effects after ICV injection can be mimicked by bilateral infusions of PACAP38 locally into the PVN. Moreover, intra-PVN administration of PACAP38 potentiated the plasma ACTH response to swim stress. This effect is likely mediated by direct activation of CRF neurons within the PVN indicated by the localization of PAC1 receptors on stress-activated (c-Fos expressing) CRF-positive neurons in the medial parvocellular part of the PVN. Thus, these findings are consistent with previous work indicating a prominent role of PACAP in stress function and extend our understanding of how this neuropeptide may influence stimulation of HPA axis activity during acute stress exposure.

These series of experiments comprehensively characterize the effects of central PACAP administration on neuroendocrine and behavioral response to a well-established stressor, forced swimming in rats. This stress paradigm enables the elucidation of neuronal mechanisms underlying stress coping strategies adopted by animals that are exposed to an inescapable stressor [50, 51]. In the present study we used forced swim challenge with one single stress exposure to avoid any potential confounding effects of learning/memory processes associated with a pre-test session as e.g., in the traditional Porsolt's-test [53]. Thus, the stress component should be emphasized in order to assess the behavioral and physiological reactivity of rats to pharmacological manipulations of the central PACAP/PAC1 receptor system. Our findings show that ICV administration of PACAP38 significantly reduced active coping and increased floating behavior indicating a more passive coping style during forced swimming, which is often interpreted as behavioral despair [57]. However, in a previous study in Wistar rats ICV PACAP38 administration failed to modulate the behavior of rats exposed to a single forced swim exposure [58]. Beside strain-specific characteristics, a possible reason for this discrepancy could be related to the use of different experimental conditions such as water depth and temperature. Notably, it is well known that increasing the water temperature reduces activity and increases the immobility time of rats in this test [59, 60] which reduces the chances of observing a further increase of immobility by the drug. Indeed, a closer look at the results from the Seiglie’s study conducted at a water temperature of 24 °C show a much higher baseline immobility time of almost 80% (for the whole 15 min exposure) and about 60% in the first 5 min of the 15-min swim session (compared to our data indicating a floating duration of 10–20%). Thus, a ceiling effect is conceivable, that might obscure the ability to see any exacerbation of floating behavior following PACAP administration. Another explanation for the discrepancy may be differences in PACAP dosage and the timing of experiments after drug administration. However, although the used dose of 0.7 µg per animal in our study vs 1–3 µg in Seiglie’s study, were slightly lower, it might have higher efficacy through an inverted U-shaped dose-effect that is quite common for neuropeptides. For example, in a previous study in rats the lower 0.3 µg dose of PACAP38 was more effective in inducing anxiety-like behavior than the 1 µg one [61].

Our data indicating that an enhanced PACAP transmission in the brain is associated with a more passive stress coping style (increased immobility) are in line with previous findings demonstrating a remarkable shift from active (burying) to passive (withdrawal) coping strategies in a shock-probe fear/defensive burying test after central PACAP administration [62]. Moreover, previous studies in rats have also shown that ICV administration of PACAP significantly increased anxiety-related behavior in approach-avoidance conflict tests such as the elevated plus maze test [10, 63] and a reduction of social interaction, also indicative of increased anxiety [58, 64]. Conversely, PACAP-deficient mice show reduced anxiety-related behavior [21, 25, 65, 66], a decreased immobility time during the first day of the traditional Porsolt’s swim test [21] and a diminished immobility response to chronic stress indicating an enhanced behavioral resiliency [67, 68] compared to wildtype mice. These data confirm that endogenous PACAP promotes anxiety-like behavior and passive coping to aversive and stressful stimuli. However, it should be noted that the opposite phenotype was also observed in other studies using different animal breeding on other genetic background [20, 65]. Thus, the effect of PACAP deficiency is also a function of the genetic mouse strain used [21].

To elucidate which brain regions may mediate the effects of PACAP we examined whether ICV infusions of PACAP affects the swim stress-induced neuronal activity in brain areas associated with stress and anxiety function such as the hypothalamic PVN and selected limbic areas. Immunohistochemical analysis of c-Fos brain mapping revealed that central PACAP infusions potentiated the swim stress-induced neuronal activity specifically within the parvocellular part of the PVN, the key region of the HPA axis regulation. Moreover, in this latter region, central administration of PACAP38 induced the expression of c-Fos even under basal conditions. This finding is consistent with previous studies in rats showing increased c-Fos-immunoreactivity in the PVN following ICV PACAP injection under stress-free (basal) conditions [9, 48], as well as after fear-conditioning [69]. On the other hand, PACAP deficiency has been shown to eliminate or considerably attenuate neuronal activation of the PVN after stressors such as restraint and forced swim [22, 23, 65, 70] suggesting that endogenous PACAP plays an important role in stress-induced activation of this nuclei and indicate the PVN as a key region mediating PACAP stress functions.

In addition to the PVN, we found an exaggerated PACAP-induced c-Fos response to forced swim stress also in discrete subregions of the LS and BNST. For example, the LS consists of a number of subregions (e.g., dorsal, medial and ventral part) defined by their neuronal cytoarchitecture and connections with other areas [71, 72]. Subsequently, subregional differences in neural activation within the LS may correspond to diverse or even opposing functions. However, PACAP increased stress-induced c-Fos expression in the ventral part of the LS an area which has been shown to be activated in response to various stressful stimuli including forced swim stress [73–75] that is strongly involved in regulating passive and fear-related responses to stress [71, 76]. Similarly, a hyperactivation of the ventral LS after stress exposure was found in rats selectively bred for high levels of anxiety [77] where stress-induced neural activation correlated with higher anxiety behavior [78]. Beside the LS, we found a c-Fos hyper-response to forced swim in other limbic pathways such as the dorsal BNST a brain region which has been shown to play a central role in regulating autonomic, neuroendocrine and behavioral stress responses [79, 80] and that may account for the mediation of stress-related behavioral effects of PACAP in the brain [70, 81].

Another candidate that may contribute to the mediation of stress-related effects of PACAP in the brain is the amygdala, where PACAP fibers and PAC1 receptors are highly expressed [5–7]. However, in contrast to an involvement in fear-related behavior [69] we found no significant differences in swim stress-induced c-Fos expression in the amygdala between PACAP-treated rats and controls, neither in the central part nor in other amygdala subregions. This was somewhat surprising given that the amygdala and especially the central amygdala has been implicated in mediating affective states of PACAP [12, 15, 18, 61, 62, 82]. However, differences in c-Fos reactivity along the anterior-posterior axis as shown for the basolateral amygdala [83] might be a reason why we found no difference in this area. Thus, quantification of c-Fos over several points along the anterior-posterior axis of the amygdala might identify PACAP-related differences in anatomically distinct neuronal subpopulations.

### Role of PACAP in the PVN in behavioral and neuroendocrine stress function

Since PACAP-immunoreactive nerve fibers [5, 42], as well as PAC1 receptors are particularly abundant in the PVN [45, 46, 84] we wanted to find out whether the PVN is directly involved in mediating PACAP effects on stress functions. Therefore, we administered PACAP38 locally into the PVN and investigated the effects of PACAP receptor modulation on the behavioral and neuroendocrine stress response. Local administration of PACAP38 into the PVN significantly increased stress-induced ACTH plasma levels, without affecting basal levels suggesting that an enhanced PACAP neurotransmission in the PVN is associated with an exaggerated neuroendocrine stress response. In the PVN, CRF neurons represent the key elements for the regulation of neuroendocrine and behavioral stress responses [39, 85–87]. So far, a PACAP-induced activation of the HPA axis of rats indicated by increased CRF expression and/or augmented plasma corticosterone levels was observed after ICV administration under basal (unstressed) conditions [9, 10, 47, 49]. Hence, our data corroborate and extend these findings by showing that elevation of PACAP transmission in the PVN increased ACTH responses to swim stress and identified the PVN of the hypothalamus as a primary site where PACAP regulates HPA responsiveness to acute stress. However, as we could not observe any differences in basal ACTH levels between PACAP-treated animals and controls our data suggest that the facilitatory influence of PACAP on basal HPA axis activity is mediated by PACAP signaling in other, probably extrahypothalamic areas. Possible candidate areas might be the BNST and central amygdala as in both areas PACAP has been shown to increase corticosterone basal levels after local administration [61, 88]. Beside central mechanisms a direct stimulatory effect on pituitary corticotropes which has been shown for PACAP [2, 89] could also be responsible for increased basal ACTH levels. Thus, our finding of unchanged basal ACTH levels but augmented ACTH levels under stress conditions in PACAP-treated rats compared to controls suggest that hypothalamic PACAP system does not regulate basal HPA axis activity but has a facilitatory role on the HPA axis responses to aversive and stressful stimuli such as forced swimming. This is in line with previous observations in transgenic PACAP-deficient mice displaying a blunted HPA axis response to primarily psychogenic stressors such as restraint and novel environment [22, 23, 31, 68, 70, 90] suggesting that endogenous PACAP plays an important role in driving HPA stress responses. We next wanted to investigate the mechanism how PACAP mediates a potential stimulatory effect on HPA axis responses to a specific stressor such as forced swim. Our finding of a colocalization of PAC1 receptors and c-Fos expression in CRF-positive neurons in the medial parvocellular PVN, suggests a direct activation of hypophysiotropic CRF neurons by PACAP which is supported by a previous electron microscopic study demonstrating that PACAP-positive terminals densely innervate CRF neurons within the parvocellular PVN region [44]. Moreover, our finding from quantification of confocal immunofluorescence data demonstrating a higher amount of CRF neurons co-expressing c-Fos after central PACAP38 administration (see Fig. 6) strengthen our conclusion that PACAP’s effects on stress mechanisms are likely mediated by the direct activation of CRF neurons within the PVN.

In addition to the modulatory effect on the neuroendocrine stress response, intra-PVN administration of PACAP38 also shifted the behavioral stress coping during forced swimming to a more passive strategy, mimicking the behavioral effects of ICV injections. Thus, these effects are in line with previous reports of stress-like behavioral actions of PACAP injections such as excessive self-grooming (e.g., face washing and body grooming) and suppressed exploratory activity [13]. Moreover, in a study evaluating the role of PACAP in modulation of fear responses it was shown that local administration of PACAP into the amygdala exert a distinct reorganization of stress-coping behavior from active (burying) to passive strategies (such as withdrawal and immobility) [62]. Thus, these data show that PACAP signaling considerably contributes to the regulation of multiple responses elicited by stress and suggest that PACAP influences stress-induced behaviors through its activity within the PVN and limbic areas such as amygdala and BNST (see also [70]). However, hypothalamic PACAP signaling is not only implicated in stress function, but also in other homeostatic activities such as glucose metabolism and body temperature regulation (for review see [91]). For instance, PACAP-injected specifically into the ventromedial nucleus or PVN has been shown to increase plasma glucose via enhanced hepatic glucose production [92, 93]. Thus, it is conceivable that catabolic effects of exogenous PACAP that increase the mobilization of energy stores also have an influence on the behavioral stress coping during forced swim test. However, as increased plasma glucose concentrations were observed 60 min following PACAP38 administration of food restricted rats a direct impact of complementary effects of PACAP as homeostatic regulator on behavioral results in our study seems very low or even negligible.

An obvious next question to answer is: which receptors mediate the behavioral effects of PACAP in the PVN? However, the absence of suitable potent and selective receptor antagonists complicates this quest. Previous studies demonstrating a significant blockade or attenuation of PACAP-induced behavioral effects (e.g., anxiogenic effect) after intracerebral administration [58, 63, 94] used the unspecific PAC1 receptor antagonist PACAP(6–38), which can not discriminate between PAC1 and VPAC2 receptors [95, 96]. Given the limitations of these studies in attributing observed behavioral effects to a specific receptor type, further studies utilizing specific antagonists are necessary to elucidate the precise role of hypothalamic PAC1 receptors in stress and anxiety functions.

Concerning possible cellular mechanisms by which PACAP mediates the observed behavioral effects in the PVN, our finding of the existence of PAC1 receptors on swim stress-activated CRF neurons within the medial parvocellular part of the PVN points to an involvement of CRF neurons. It is now well accepted that PVN-CRF neurons are not only implicated in regulating the neuroendocrine but also behavioral stress response (see above). Indeed, PACAP-induced anxiogenic and anhedonic-like behavioral effects in rats could be blocked by pre-administration of a selective CRF receptor antagonist [10] suggesting the CRF system as an immediate downstream target and mediator of PACAP effects. Beside CRF there are also other potential candidates for mediating PACAP-induced stress responses. Consistent with previous neuroanatomical studies [45, 46] our immunohistochemical data show an abundant expression of PAC1 receptors not only in the parvocellular but also in the magnocellular subdivision of the PVN (see Fig. 5). Thus, it is conceivable that vasopressin and oxytocin-producing magnocellular neurons that seem also to play a role in behavioral stress function [97, 98] may be implicated in mediating PACAP effects in the PVN. Indeed, PACAP has been shown to increase the expression of vasopressin in the magnocellular part of the PVN [48] and can modulate the function of these neurons by increasing their firing rate [99]. However, our finding of a significant difference of swim stress-induced c-Fos expression in the parvocellular, but not magnocellular PVN of PACAP-treated animals compared to controls contradicts this assumption. Nonetheless, as both vasopressin and oxytocin are also expressed in parvocellular neurons of the PVN a contribution of these neuropeptides in mediating PACAP effects can not completely excluded. Especially, vasopressin is of particular interest because of its well-documented synergistic effect with CRF on ACTH secretion. In addition, beside neuropeptides, in particular CRF neurons in the PVN co-express the major amino acid neurotransmitters glutamate and GABA that are known to play a major role in stress function [40, 41, 100]. Therefore, further studies are needed to elucidate the exact underlying neural mechanisms that mediate PACAP-induced behavioral effects within the hypothalamic PVN. Thereby novel techniques (e.g., optogenetic and chemogenetic approaches) that allow for the targeting of specific neuronal populations and the use of PAC1 receptor-selective antagonists will be useful in these investigations.

A limitation of the present study is that only male animals were used. A growing number of studies documents sex-specific aspects of PACAP signaling and function [101, 102]. For example, changes in PACAP levels in brain areas such as hypothalamus following food or water deprivation stress have been shown to differ in male and female rats [103]. In humans, a single nucleotide polymorphism in the gene coding for the PAC1 receptor has been consistently related to PTSD diagnosis and symptomatology in trauma-exposed females, but not in males [34, 35]. Additionally, previous work showed that the PACAP-PAC1 receptor system is important for fear extinction in highly traumatized women and a PTSD-like model in female mice that exhibit a PACAP upregulation in hypothalamic areas [104]. Thus, while we observed strong behavioral effects in males, it is possible that PACAP effects may be more pronounced in females, which will be a focus of future research endeavors.

In summary, the present data show that the PACAP/PAC1 receptor system plays a critical role in modulating neuroendocrine and behavioral responses to acute stress. Particularly, centrally administered PACAP promotes passive coping responses during forced swimming indicative of a maladaptive behavioral stress coping that can be mimicked by local infusion of this peptide into the hypothalamic PVN. Additionally, these animals show a disinhibited neuroendocrine stress response that is mediated probably through direct activation of CRF neurons via PAC1 receptors within the PVN itself. Thus, we identified the PVN as a critical site where PACAP regulates the behavioral and neuroendocrine stress response. Moreover, the present findings suggest that exaggerated PACAP transmission prevents animals to cope adequately with a stressful experience and strengthen the view that certain stress- and trauma-related psychiatric disorders are related to an upregulation of the PACAP/PAC1 receptor system in the brain [105, 106]. Consequently, elevated PACAP levels might be a useful transdiagnostic biomarker for the severity of psychiatric symptoms [35, 36, 38] and pharmacological blockade of PACAP signaling via selective receptor antagonists may represent a potential novel avenue for the treatment of these stress-related psychiatric disorders.

## Supplementary information

Suppl. Mat

## Data Availability

Data will be made available on request.
