# Supplementary material for: PACAP regulates neuroendocrine and behavioral stress responses via CRF-containing neurons of the rat hypothalamic paraventricular nucleus
Source: Neuropsychopharmacology. 2024 Oct 29;50(3):519–30. doi: 10.1038/s41386-024-02016-9 (PMC11735793; doi:10.1038/s41386-024-02016-9)
Supplement: Supplementary file 1 — Suppl. Mat [file 41386_2024_2016_MOESM1_ESM.pdf]

Supplementary Materials

Material and Methods

Animals

Experiments were carried out on adult male Sprague-Dawley rats (250-350 g). Prior to use, the animals were housed in groups of 2-4 under controlled laboratory conditions (12:12 h light/dark cycle with lights on at 7:00 a.m., 22 ± 2°C, 45-65 % humidity, pelleted food and water ad libitum) for at least one week after delivery from the supplier (Charles River Laboratories, Sulzfeld, Germany). Experiments were performed between 08:00 and 13:00 h in accordance with the guide for the care and use of laboratory animals and approved by the Austrian Animal Experimentation Ethical committee (Bundesministerium für Wissenschaft, Forschung und Wirtschaft, Kommission für Tierversuchsangelegenheiten).

Surgery

All surgical procedures were performed under sterile conditions. Prior to surgery rats were anaesthetized with isoflurane (as inhalant: 5% induction; 2.5 % maintenance) by using a vaporizer system (EZ systems corporation, Palmer, USA). To prevent hypothermia during surgeries, animals were placed on a heating pad. Following surgery, animals received post-operative analgesia by injection of buprenorphine (0.5 mg/kg s.c.; 3 x every 8 hrs; Richter Pharma AG, Austria) and meloxicam (1 mg/kg p.o. via drinking water, Boehringer Ingelheim, Germany) and were housed individually during postsurgical recovery period. The rats were monitored by daily weighing and handling, including removal of the dummy cannula in order to habituate animals to subsequent intracerebral injection procedure, thereby minimizing the stress reaction on the experimental day.

Stereotaxic surgery

The stereotactic surgical procedures were performed as previously described [1,2]. Briefly, anesthetized animals were fixed in a stereotaxic frame (Stoelting, Illinois, USA) and a 23-gauge stainless steel guide cannula (15 mm length; o.d. 0.64 mm, i.d. 0.34 mm; Injecta GmbH, Germany) was implanted either unilaterally 1 mm above the right lateral ventricle (coordinates from bregma: AP 0.8 mm, ML +1.4 mm and DV -3.0 mm) or bilaterally 2 mm above the left and right PVN (coordinates from bregma: AP 1.9 mm, ML +/- 1.7 mm, DV -6.3 mm, with an angle of 10°) by using a rat brain atlas [3]. Guide cannulas were fixed to the skull via two stainless steel screws and dental acrylic cement (Dentalon plus, Heraeus Kulzer GmbH, Germany). Thereafter, a dummy stylet was inserted into each guide cannula to prevent clogging and

reduce the risk of infection. Rats were allowed a recovery period of 5-7 days before experiments. The locations of the cannula track were histologically verified after experiments (see Figure 3).

Implantation of a jugular venous catheter

A silastic-tipped vinyl catheter was inserted into the left jugular vein, routed under the skin and exteriorized at the neck of the animal as described previously [2]. The wounds were closed using metal clips. The catheter was filled with sterile saline containing gentamycin (30 000 IU/rat; Centravet, Bad Bentheim, Germany) and flushed with the same solution 2 days after surgery. On the day of the experiment, the catheter was connected to a 1-ml plastic syringe via an approximately 40-cm long piece of polyethylene-50 tubing (Becton Dickinson, Sparks, USA) 2 h before starting the experiment. Blood sampling through a pre-implanted jugular venous catheter allows repeated blood sampling from conscious, freely-moving rats without restraining animals.

Forced swim challenge

The forced swim challenge was conducted as described previously [2,4]. Briefly, rats were placed individually in a square plastic tank (35 x 35 cm) filled with water (20°C ±1°C) to a 30-cm depth. During the forced swimming session, the behavior was recorded by a video system for subsequent analysis. Animal's behavior was scored by a trained observer blind to the treatment of animals, quantifying absolute time measurements. The behavior of the animals was assigned to one of the three following behavioral categories: (1) struggling, defined as movements during which the forelimbs broke the surface of water; (2) swimming, defined as movement of the animal induced by movements of the fore and hind limbs without breaking the water surface; and (3) floating defined as the behavior during which the animal used limb movement just to keep its equilibrium without any movement of the trunk [2,4]. After the 5-min swimming session, animals were gently dried using a towel and returned to their home cage.

Drug microinjection procedure

For ICV infusions, rats were gently held inside their cages and the stylets of guide cannulas were removed. Drugs were injected into the right lateral ventricle (1.5 µl/rat) over a period of approximately 1 min using a 5-µl syringe (Hamilton Instruments, Switzerland) that was connected via a polyethylene tubing to a 30-gauge injection cannula (tip extending 1 mm beyond the guide). The internal cannula was held in a position for another 2 min after injection

to allow diffusion of drugs before being slowly withdrawn. Rats were returned to their home cage and 15 min after the injection exposed to the modified forced swim test.

For bilateral microinfusions into the PVN, stylets of guide cannulas were replaced by two 30-gauge microinjection cannulas that were 2 mm longer than the guide cannulas, thus reaching the PVN. Injection cannulas were connected to a 5 cm long PE-10 tubing and filled with drug or vehicle solution. This infusion device was connected to a syringe mounted on a microinfusion pump (TSE-Systems, Bad Homburg, Germany) via a 100 cm long polyethylene tubing interconnected with a dual channel fluid swivel system (Instec Laboratories, Boulder, USA). Dugs were infused over a period of 7.5 min at a defined flow rate of 0.2 µl/min (1.5 µl/injection side). After the microinjection procedure, the injection cannula was left in the guide cannula for another 2.5 min before being removed. Thereafter, animals were exposed to the forced swim stressor.

Blood sampling and ACTH measurements

After a 60-min habituation, the experiment started with the collection of two blood samples (0.3 ml) under basal conditions, 35 and 15 min prior to stress exposure. Bilateral infusions of drugs into the PVN started 12 min before the onset of the stressor. After injections, another blood sample was taken and 1 min later animals were exposed to the forced swim stress procedure for 5 min. During the forced swim session, behavioral output was scored and analysed as described above. After animals were returned to their home cages three additional blood samples were collected at regular intervals (10, 30, and 60 min after the onset of the stressor). Sampled blood volumes were immediately replaced with an equal volume of heparinized saline. Blood was collected into EDTA-coated tubes containing 10 units of sodium heparin (Wako Pure Chemical Industries Ltd., Osaka, Japan), placed on ice and immediately centrifuged at 3000 g for 10 minutes at 4°C. Supernatants were collected and stored at −80°C until measurement. Plasma ACTH concentrations were determined using a commercially available immunoassay kit (MP Biomedicals, Orangeburg, NY, USA) according to the manufacturer's protocol. The intra- and inter-assay coefficients of variation were below 7 and 10 %, respectively.

Drugs

PACAP38 (Bachem AG, Switzerland) were dissolved in sterile distilled water and aliquots of stock solution (1 mg/mL) were stored at -80°C. For preparation of the working solution the liquid of concentrated stock solution was diluted with artificial cerebrospinal fluid (aCSF; 140 mM NaCl, 3.0 mM KCl, 1.25 mM $CaCl_2$, 1.0 mM $MgCl_2$, 1.2 mM Na_2HPO_4, 0.3 mM NaH_2PO_4,

3.0 mM glucose and pH adjusted to 7.4) to a final concentration of 10 or 100 µM. Vehicle animals received a 1.5 µl infusion of aCSF solution. All drugs were freshly prepared before each experiment and kept on ice during the experimental procedures.

Histological verification of cannula placements

At the end of experiments, the animals were sacrificed by an overdose of sodium thiopental and brains were rapidly removed, snap-frozen in 2-methylbutane solution and stored at -20°C. For histological verification of the placement of microinjection cannulas within the lateral ventricle or PVN, brains were sectioned (40 µm coronal sections) using a cryostat (Leica CM1950, Leica Microsystems, Germany). Brain sections were mounted on gelatin-coated slides (Carl Roth, Germany) and stained with cresyl violet. Verifications of cannula placements were made before analyzing neuroendocrine and behavioral experiments under a microscope on the basis of previous definitions of a brain atlas [3]. Only data from rats found to have injector tracks extending into appropriate target sites were included in the analyses.

Perfusion and brain tissue processing

Two hours after stress exposure, animals were deeply anesthetized with sodium thiopental (250 mg/kg i.p., Sandoz, Austria) and transcardially perfused with 300 ml of 0.9% saline followed by 300 ml of fixative (either 4% paraformaldehyde in 0.1 M PBS or 2% paraformaldehyde with 0.2% picric acid in 0.1 M PBS, Stefanini´s solution). Brains were removed and kept in fixative at 4°C overnight. After post-fixation, the brains were washed twice in 0.2 M PBS (c-Fos immunohistochemistry) or equilibrated in 0.05 M PBS containing 30% sucrose for 48 hours at 4°C (Immunofluorescence staining) until further processing. Series of 40 µm-thick coronal sections of respective brain areas were sectioned by using either a vibratome (Leica VT1000S, Leica Microsystems, Germany) or a cryostat (Leica CM1950, Leica Microsystems, Germany). The sections were stored in a cryoprotectant solution at 4°C until further processing.

c-Fos staining by DAB immunohistochemistry

Brain sections were processed for c-Fos immunoreactivity as described previously [1]. Briefly, sections were incubated in 1% hydrogen peroxide in tris-buffered saline (TBS, pH=7.4) for 30 min, followed by three washes with TBS for 10 min each and incubated in blocking solution using 1% bovine serum albumin (BSA) in 50 mM TBS containing 0.1% Triton-X-100 (all from Sigma-Aldrich). Sections were incubated with the rabbit anti c-Fos primary antibody (1:2000;

sc-52, Santa Cruz Biotechnology) at room temperature in a humid chamber for 48 hours. Subsequently, sections were incubated with the corresponding biotinylated goat anti-rabbit secondary antibody (1:500; Vector laboratories) at room temperature for 24 hours. The formed antigen-antibody-complex was visualized by the avidin-biotin-horseradish peroxidase procedure (Vectastain ABC kit; Vector Laboratories) using 3,3'-diaminobenzidine (DAB) as the chromogen. Cells containing a nuclear brown-black DAB staining were considered as c-Fos positive cells. The reaction was terminated once an optimal contrast between specific cellular and nonspecific background labeling was reached. Immunoreactive cells were quantified manually in the regions of interest by a trained experimenter blind to the treatment groups using a brightfield microscope (Olympus BX-51, Olympus Corporation) equipped with a computer-assisted image analysis system. Sections from all investigated animals were chosen at identical rostro-caudal levels, making direct comparison between animals possible. Subregions of interest (e.g. parvocellular/magnocellular subdivision of the PVN) were selected on neurochemical and functional criteria. For instance, we specifically chose brain sections from the medial parvocellular part of the PVN, as this sub-region is known to be enriched in CRF-expressing neurons, which are highly relevant to our study. Anatomical localization of selected brain regions was aided by using illustrations of a rat brain atlas [3].

Immunofluorescence staining

Immunofluorescence staining was performed as previously described [5]. Briefly, selected brain sections of the PVN region were incubated in antigen retrieval buffer (DAKO, ChemMate, code No. S203120) diluted in distilled water (1:1. pH=6) for 90 min at 80°C (pre-heated in a microwave) before processing for immunohistochemistry (described above). Brain slices were washed with PBS/0.25% Triton X-100 (Sigma-Aldrich) and incubated in 1% hydrogen peroxide in PBS (pH=7.4) for 20 minutes to quench endogenous peroxidase activity. Brain slices were washed with PBS/0.25% Triton X-100 (3 x 10 min/wash) and incubated in 5% normal donkey serum (Jackson ImmunoResearch) diluted in PBS/0.25% Triton X-100/bovine serum-containing albumin (Sigma-Aldrich) for a further 30 minutes at room temperature. Successively, sections were incubated in a solution containing rabbit anti-PAC1 antibody (Code 35J8, RRID: AB_2814675, diluted 1:10000) overnight at 4°C. After rinsing in PBS/0.25% Triton X-100 (3 x 10 min/wash), sections were incubated in DAKO-ENVISION system HRP (DAKO K003, Agilent Technologies, USA), diluted 1:2 in PBS/0.25% Triton X-100 and/bovine serum albumin (Sigma-Aldrich, USA) overnight at 4°C. On the third day, sections were washed in PBS/0.25% Triton X-100 (3 x 10 min/wash) and incubated in Alexa Fluor™ 647 Tyramide Reagent (ThernoFisher scientific, B40958) diluted 1:200 in amplification buffer (PerkinElmer, FP1052) for one hour at room temperature. Hereafter sections were washed and incubated in

a solution containing a mixture of a rabbit anti-CRH antiserum (H-019-06, Phoenix Pharmaceuticals, Inc., 1:500) and a mouse anti-c-Fos (c-10) antibody (sc-271243, Santa Cruz Biotechnology, 1:500). On day four, brain sections were washed and incubated in a solution containing a mixture of Alexa 488 conjugated donkey-anti rabbit (1: 400, # 711-545-152, Jackson ImmunoResearch) and Texas red 594 conjugated donkey anti-mouse antibodies (1:200, #715-075-151, Jackson ImmunoResearch). On day five, brain sections were washed with PBS/0.25% Triton X-100 (3 x 10min/wash) and mounted on Superfrost Plus Menzel-Gläser (ThermoFisher Scientific, J1800AMNZ) in a 0.5% gelatine medium (Sigma, G2500). When the sections were dried, slides were coverslipped with DAPI (Molecular Probes, D-1306) dissolved in glycerol (Sigma-Aldrich, G9012) as a nuclear counterstain.

Photomicroscopy was performed using an iMIC confocal microscope equipped with the following objectives: X10, numerical aperture (NA) = 0.35; X20, NA = 0.75; X40, NA = 1.3 and X60, NA = 1.46. Using the X60, the highest resolution (($r = \lambda/NA$), where λ is the imaging wavelength) was for X60 = 174 nm. The resolution in the z-axis was at X60 0.2 μm. For 3D analysis images in Z-stacks photographed using the X40 or X60 objective were deconvoluted in AutoQuantX, version 3.04 (Media Cybernetics, Inc. Rockville, USA) before analyzed in IMARIS® vers. 8.4.1 (RRID: SCR_007370) from Bitplane, Switzerland (http://www.bitplane.com). All images were adjusted for brightness and contrast either in Fiji or in Photoshop CS5 (Adobe, San Jose, CA, RRID: SCR_014199) and mounted into plates in Adobe Illustrator CS5 (Adobe).

Quantitative immunohistochemistry

Quantification was performed using the cell counter module in Fiji software (version 1.47q, NIH, USA, RRID:SCR_003070) for counting CRF and FOS expressing cells as described previously [6]. Quantification of PAC1 receptor expression in the mcPVN and mpcPVN were determined in Fiji software (one sections from the mid PVN of each of 3 saline and - 3 PACAP38 treated animals were used) after calibration using 8-bit gray tone images obtained using identical settings in the microscope (exposure time, gain) by the confocal unit and X20 objectives and calibrated optical density (O.D.) as described (https://imagej.nih.gov/ij/docs/examples/calibration/) [7].

Statistical analysis

Experimental subjects were included in the statistical analysis only if the microinjection cannulas were confirmed to be localized in the respective brain region. Statistical analysis was performed using GraphPad Prism 5. Counts of c-Fos positive nuclei within the different brain

areas and behavioral data after ICV drug injection were analyzed using parametric Student's two-tailed t-test. Behavioral data from more than two groups were analyzed by one-way ANOVA followed by Dunnet's multiple comparison post hoc test. Plasma concentrations of ACTH were analyzed by two-way ANOVA (treatment x time) with repeated measures on the last factor followed by appropriate post hoc analysis. Data are presented as mean ± standard error of the mean (SEM). In all cases, $P<0.05$ was considered to be statistically significant.

References

1. Ebner K, Muigg P, Singewald N. Inhibitory function of the dorsomedial hypothalamic nucleus on the hypothalamic-pituitary-adrenal axis response to an emotional stressor but not immune challenge. J Neuroendocrinol. 2013;25(1):48-55.

2. Singewald GM, Rjabokon A, Singewald N, Ebner K. The modulatory role of the lateral septum on neuroendocrine and behavioral stress responses. Neuropsychopharmacology. 2011;36(4):793-804.

3. Paxinos G, Watson G. The Rat Brain in Stereotaxic Coordinates. Academic Press; 2007.

4. Ebner K, Singewald GM, Whittle N, Ferraguti F, Singewald N. Neurokinin 1 receptor antagonism promotes active stress coping via enhanced septal 5-HT transmission. Neuropsychopharmacology. 2008;33(8):1929-41.

5. Hannibal J, Georg B, Fahrenkrug J. PAC1- and VPAC2 receptors in light regulated behavior and physiology: Studies in single and double mutant mice. PLoS One. 2017;12(11):e0188166.

6. Riedel CS, Georg B, Hannibal J. Phenotyping of light-activated neurons in the mouse SCN based on the expression of FOS and EGR1. Front Physiol. 2023;14:1321007.

7. Riedel CS, Georg B, Fahrenkrug J, Hannibal J. Altered light induced EGR1 expression in the SCN of PACAP deficient mice. PLoS One. 2020;15(5):e0232748.

Supplemental Data

Table S1. Effects of ICV PACAP38 administration on c-Fos expression under basal conditions.

Brain Region	aCSF (controls)	PACAP38
Lateral septum		
Lateral septal nucleus, dorsal	2.7 ± 0.4	3.7 ± 0.6
Lateral septal nucleus, ventral	7.8 ± 1.2	7.1 ± 0.8
Bed nucleus of the stria terminalis		
latero-dorsal part	6.0 ± 0.9	6.3 ± 0.5
latero-posterior part	4.8 ± 0.4	4.9 ± 0.6
medial-anterior part	3.2 ± 0.5	4.0 ± 0.2
Paraventricular hypothalamic nucleus		
parvocellular part	4.4 ± 0.5	8.3 ± 0.8 *
magnocellular part	2.8 ± 0.4	4.2 ± 0.7
Amygdala		
Central nucleus of the amygdala	7.0 ± 1.5	7.2 ± 1.0
Basolateral nucleus of the amygdala	3.9 ± 0.5	4.0 ± 0.5
Medial nucleus of the amygdala	4.2 ± 0.3	3.8 ± 0.8

Data are expressed as mean ± SEM. Values are numbers of c-Fos positive cells/0.01mm^2. N=6 animals per group. * $p < 0.05$ vs aCSF-injected controls (Student's t-test).

Supplemental Data

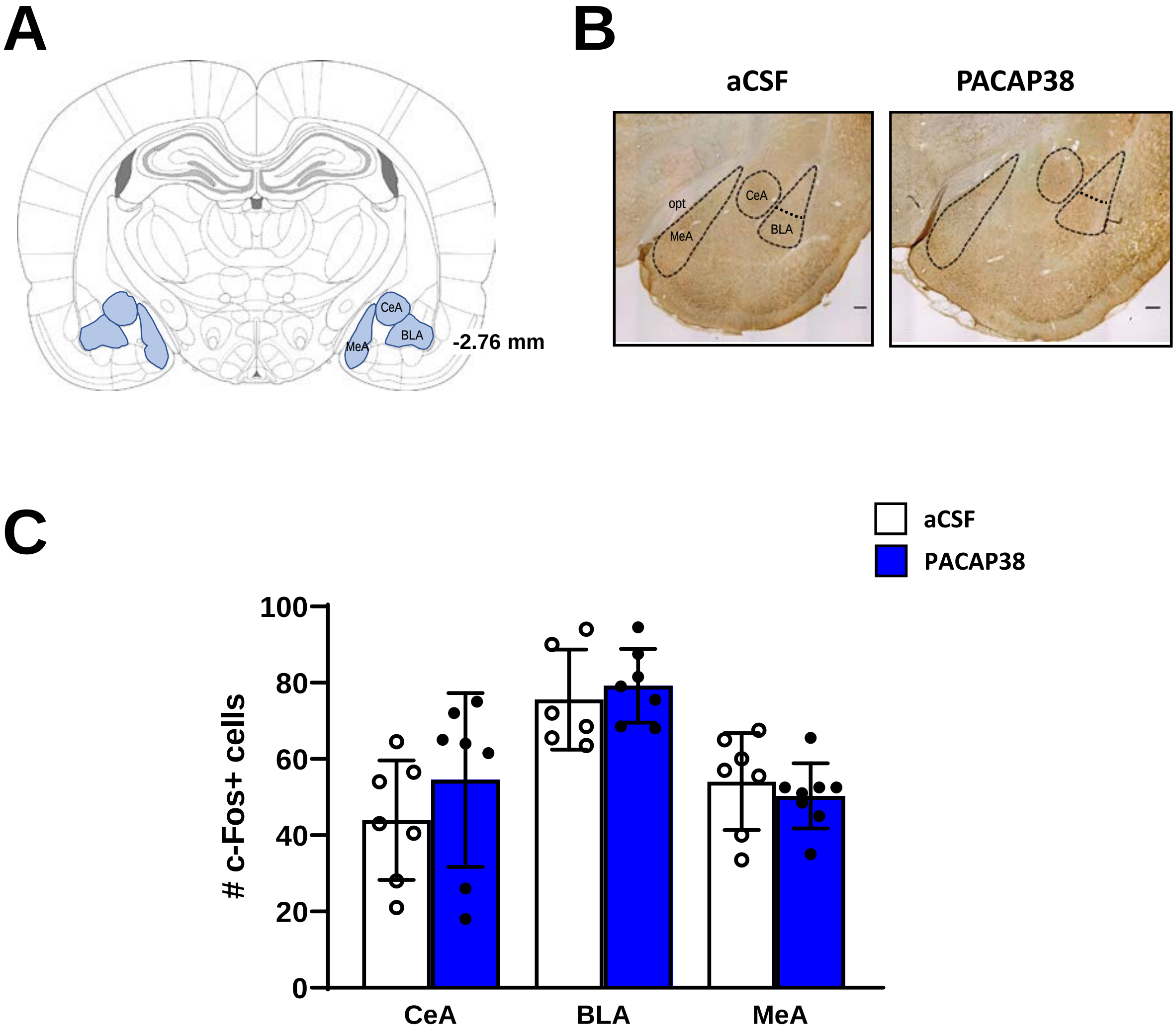

Figure S1. Effects of ICV PACAP38 administration on swim stress induced c-Fos expression in the amygdala. (A) A schematic coronal section from the brain atlas of Paxinos and Watson (2007) showing different subregions of the amygdala examined (gray shaded). (B) Representative photomicrographs showing c-Fos positive cells in rats exposed to forced swim stress and ICV microinjected either with aCSF or PACAP38. (C) The number of c-Fos immunoreactive nuclei in the medial (MeA), central (CeA) and basolateral amygdala (BLA) presented as bar graphs. There was no difference of stress-induced c-Fos expression in neither of the amygdalar subareas between PACAP38 treated animals and aCSF-injected controls. Abbreviations: AMY, amygdala; opt, optic nerve. N=6-8 animals per group. Scale bar: 200 µm. Data are expressed as mean ± SEM.

Supplemental Data

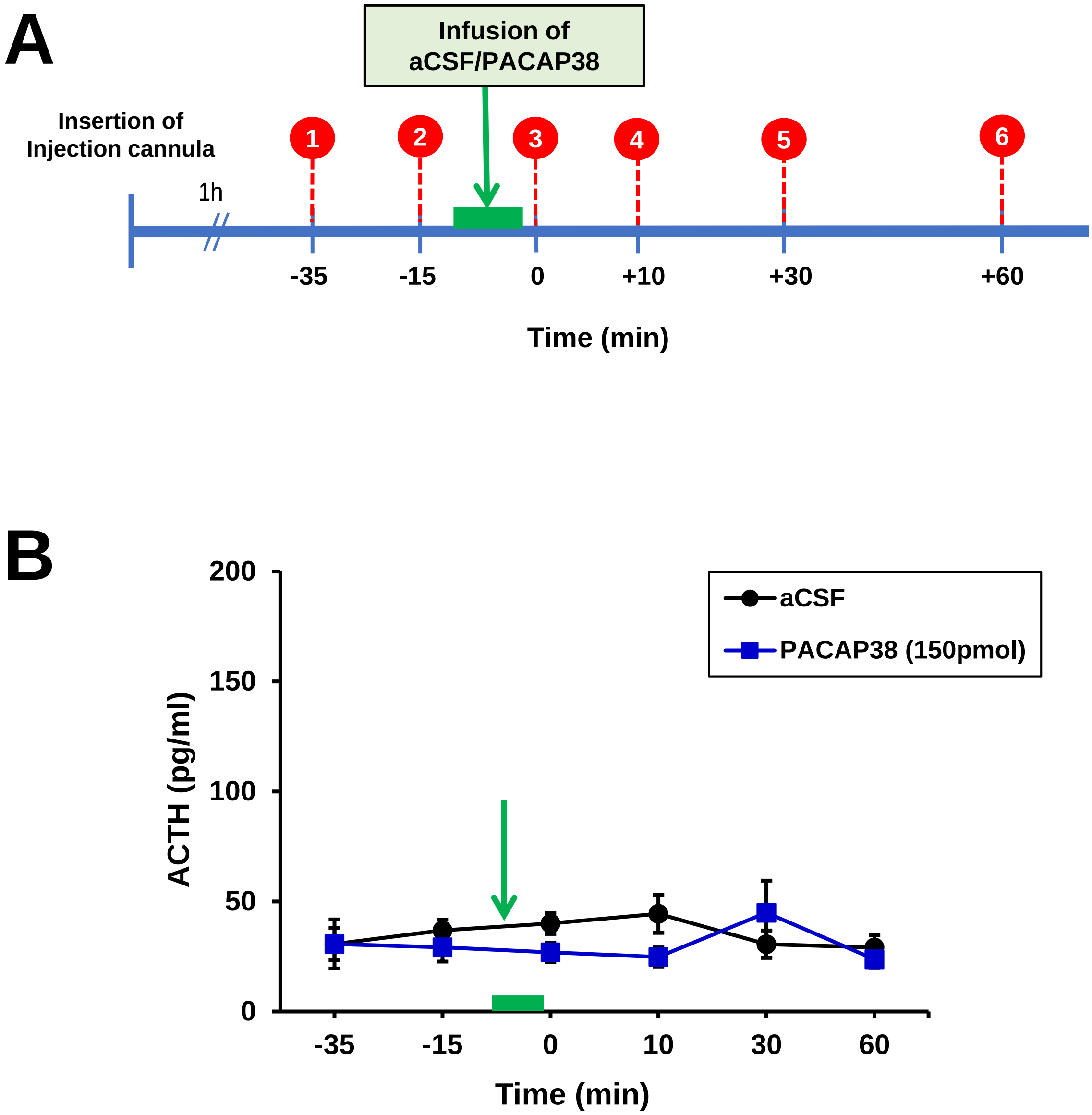

Figure S2. Effects of PACAP38 administration into the PVN on basal ACTH levels. (A) Schematic illustration of the experimental design with the timeline of blood sampling (red circles), and drug infusion (green bar). Experiment started with insertion of the infusion device (bilateral injection cannulas connected to a microinfusion pump) at least 1 h before blood sampling started. Drugs were infused automatically at a constant flow rate over a period of 7.5 min without any stressful manipulations (e.g. such as capturing or restraining animals) before and during the infusion procedure. Blood samples were collected at regular intervals before (-35 and -15 min) and after drug infusion (10, 30, and 60 min). (B) Time course of ACTH plasma levels before and after PACAP38 administration. Note that ACTH levels did not differ between groups. The green bar/arrow indicates timing of intra-PVN infusion. N= 4-5 animals per group. Data are expressed as mean ± SEM.

Supplemental Data

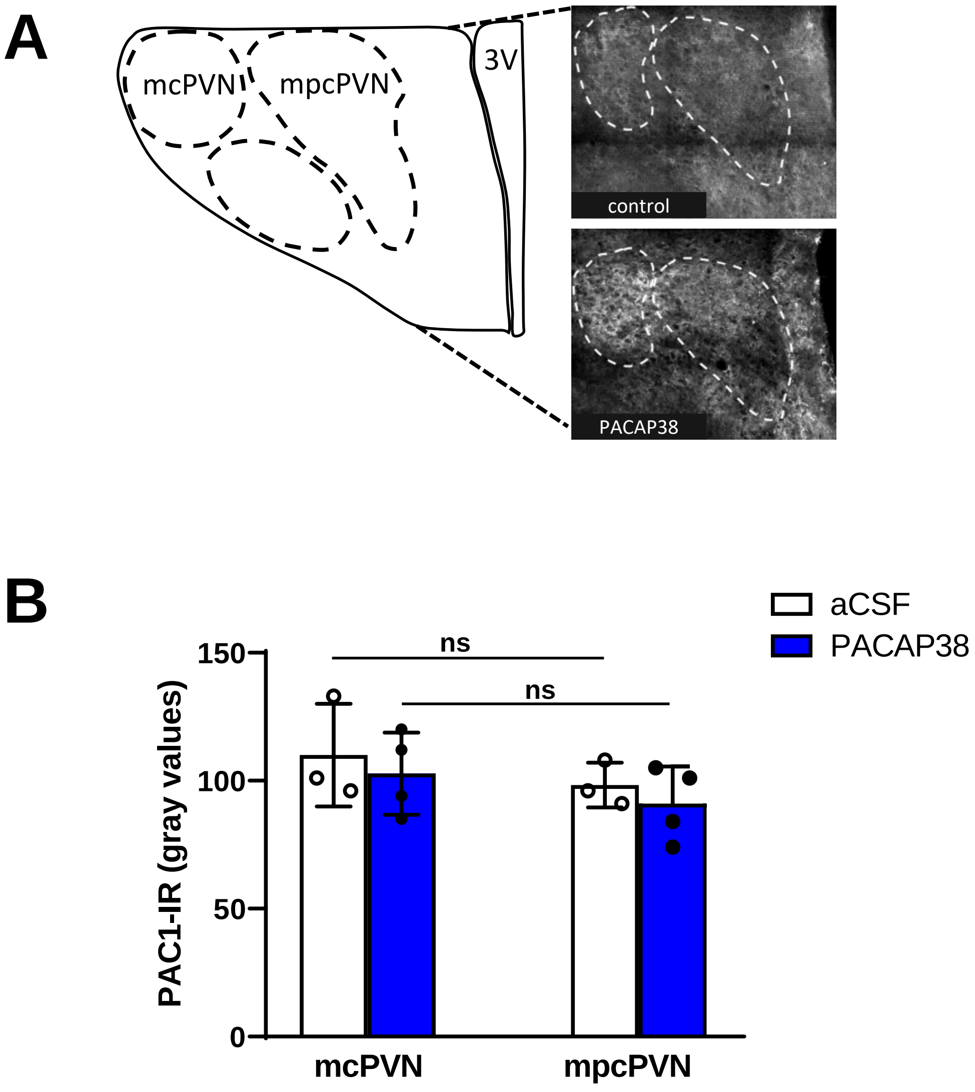

Figure S3. Quantification of PAC1 receptor immunoreactivity in the PVN of rats that were ICV injected with aCSF (controls) or PACAP38 (150 pmol) 15 min before animals were exposed to forced swim stress. (A) Schematic drawing illustrating different subregions of the PVN and representative photomicrographs showing PAC1 receptor immunoreactivity in the PVN that was determined using a confocal 8-bit gray scale images calibration. (B) Quantified PAC1 receptor immunoreactivity expressed as gray values in the in the lateral magnocellular PVN (mcPVN) and the medial parvocellular PVN (mpcPVN). There was no difference of PAC1 receptor immunoreactivity in neither of the PVN subareas between PACAP38 treated animals and controls. Note that PAC1 expression is not significant different between the mcPVN and the mpcPVN. Abbreviations: 3V, third ventricle; mcPVN, magnocellular PVN; mpcPVN, medial parvocellular PVN; ns, not significant. N=3-4 animals per group. Data are expressed as mean ± SEM.
